# Empowering rural governance with digital technology: Deep learning models for automated detection of rural buildings using remote sensing images

**DOI:** 10.1371/journal.pone.0351311

**Published:** 2026-06-12

**Authors:** Jingling Zhong, Youcai Xie, Lixia Li, Chuanlin Shi

**Affiliations:** 1 School of Managements, Guangzhou Huashang College, Guangzhou, Guangdong, China; 2 School of Artificial Intelligence, Guangzhou Huashang College, Guangzhou, Guangdong, China; 3 School of Public Administration, South China Agricultural University, Guangzhou, Guangdong, China; Chunghwa Telecom Co. Ltd., TAIWAN

## Abstract

Building detection from drone imagery represents a transformative approach to rural governance by enabling precise spatial data acquisition for critical applications including illegal construction monitoring, disaster assessment, and cadastral mapping. However, automated detection systems face persistent challenges including extreme scale variations in rural buildings, complex background interference from vegetation and shadows leading to boundary ambiguity, and severe scarcity of high-quality annotated datasets that limit model generalization. To overcome these limitations, this study introduces an integrated framework featuring three innovative components: the Multi-scale Hybrid Attention module employs parallel convolutional pathways with channel and spatial attention to dynamically capture multi-scale features while suppressing background noise; the Dynamic Feature Pyramid Network utilizes content-aware routing to adaptively fuse hierarchical features for optimal scale-invariant representation; and the Progressive Contrastive Learning strategy leverages both labeled and unlabeled data through hard sample mining to enhance discriminability under data constraints. Extensive experiments validate the model’s efficacy, achieving a mean Intersection over Union (MIoU) of 87.3%, pixel accuracy (PA) of 94.2%, and mean Average Precision (mAP) of 89.6% on the Massachusetts Buildings Dataset, substantially surpassing benchmarks like U-Net (80.1% MIoU), SegNet (78.9% MIoU), and DeepLabV3+ (82.4% MIoU), with ablation studies confirming critical module contributions (e.g., MIoU drops to 81.5% without MHA). The framework demonstrates robust cross-dataset generalization (72.3% MIoU on Chinese rural data) and effective problem resolution, establishing a scalable solution for intelligent rural governance through accurate building extraction. The dataset and code used in this study have been uploaded to the GitHub website: https://github.com/xiexie1234567890/rural_building_detection/tree/main.

## Introduction

Building detection from drone imagery plays a pivotal role in advancing rural governance by enabling a transformative shift from conventional, labor-intensive practices to data-driven, precise, and intelligent management [1]. This technology provides a foundational means of acquiring accurate, real-time spatial data on building footprints, distribution patterns, and structural conditions—information essential for multiple governance functions. It facilitates the efficient identification of unauthorized constructions, such as unpermitted buildings or those exceeding approved dimensions, thereby promoting compliance with land-use regulations and supporting evidence-based spatial planning [2]. Additionally, its application extends to evaluating structural damage and safety hazards, which is crucial for post-disaster emergency response, the management of deteriorating housing stock, and ensuring residential safety—key components of an integrated “sky-air-ground” monitoring framework [3]. Beyond regulatory enforcement and risk mitigation, the high-resolution spatial data supports critical administrative tasks including property rights verification, cadastral mapping, and digital village archiving, thus establishing a robust spatial database for sustainable resource allocation and cultural heritage preservation [4]. When integrated with Geographic Information Systems (GIS), drone-derived data enables the development of realistic 3D reconstructions and dynamic monitoring platforms, enhancing targeted interventions in rural renewal, human settlement improvement, and long-term territorial planning [5]. Ultimately, this integration fosters the realization of digitally enabled “smart villages,” advancing rural modernization through technological innovation.

Early methodologies relied on conventional classification algorithms and shallow learning models, which frequently underperformed due to the spectral heterogeneity and spatial irregularity characteristic of rural built environments in high-resolution imagery [6]. The emergence of deep convolutional neural networks (CNNs) [7], particularly fully convolutional network (FCN)-based architectures such as U-Net [8] and SegNet [9], represented a pivotal breakthrough. These models excel at pixel-level semantic segmentation by effectively integrating spectral information with rich spatial context, thereby achieving precise delineation of building footprints. Empirical studies have shown that architectures like SegNet attain high overall accuracy, substantially surpassing the performance of earlier approaches [10]. This capability establishes a critical data foundation for diverse applications in rural governance.

Nevertheless, rural scenes present distinct challenges, including small building scales, complex backgrounds composed of vegetation and farmland, and high visual similarity between partially constructed and completed structures—necessitating continuous architectural innovation [11]. To mitigate issues such as missed detections and inconsistent boundary delineation, researchers have developed increasingly sophisticated model frameworks. Hybrid architectures that integrate CNNs with Graph Neural Networks (GNNs) or Transformer modules have been introduced to capture long-range contextual dependencies and enhance feature discrimination [12,13]. Multi-task learning frameworks incorporating auxiliary boundary-aware branches and adversarial optimization strategies have been proposed to improve the geometric fidelity of detected building shapes [14]. Moreover, emerging paradigms such as contrastive learning and self-training with pseudo-label generation are being investigated to address class imbalance and reduce dependence on large-scale, manually annotated datasets [15]. Collectively, these advancements significantly improve the robustness and precision of building detection, facilitating finer-grained, data-driven approaches to rural planning and management.

There are five primary deep learning models commonly used for detecting rural buildings.

**U-Net [**16**]** employs a symmetric encoder-decoder architecture with skip connections, facilitating precise pixel-wise segmentation by combining high-resolution spatial information from the encoder with contextual features from the decoder. This design is particularly effective for detecting rural buildings, as it preserves fine-grained details of irregular building boundaries in drone imagery. Its primary advantage is achieving high accuracy with limited training data. However, the model may struggle with complex background interference and requires substantial computational resources for processing high-resolution images.

**SegNet [**17**]** utilizes an encoder-decoder framework with a unique max-pooling indices mechanism during upsampling, which reduces memory usage while retaining critical boundary information for segmentation tasks. This model demonstrates strong performance in extracting rural buildings from high-resolution remote sensing images by effectively leveraging both spectral and spatial features. Its lightweight architecture enables efficient training and inference. However, a limitation is its occasional insensitivity to small-scale structures and potential boundary inaccuracies in heterogeneous rural landscapes.

**Mask R-CNN [**18**]** extends Faster R-CNN by adding a parallel branch for pixel-level mask prediction, enabling instance segmentation of individual buildings. The integration of RoI Align replaces RoI Pooling to prevent misalignment issues, thereby improving accuracy in delineating building contours. This model excels at handling occluded or densely packed rural structures and supports multi-task learning. However, its two-stage architecture results in higher computational costs, making it less suitable for real-time applications on resource-constrained platforms.

**ResNet [**19**]** introduces residual learning through skip connections to mitigate the vanishing gradient problem in deep networks, enabling the training of very deep architectures. This backbone network improves the robustness of feature extraction for tasks such as building classification and damage assessment in rural areas. Its strength lies in superior feature representation and transfer learning capabilities. However, the increased depth of the model leads to greater parameter complexity, which can result in overfitting when annotated rural data is limited.

**YOLO [**19**]** is a single-stage detector that unifies object localization and classification into a single neural network pass, significantly accelerating inference speed. Innovations such as adaptive anchor computation and mosaic data augmentation enhance its adaptability to varying rural building sizes and orientations. It achieves an optimal balance between speed and accuracy, making it well-suited for real-time drone video analysis. However, its drawbacks include relatively lower precision for small or overlapping buildings compared to two-stage models.

Despite significant advancements in deep learning for rural building detection from remote sensing imagery, the field continues to grapple with several persistent and interconnected challenges that hinder the achievement of robust and highly accurate automated systems. A primary obstacle is the substantial scale variation and irregular distribution of rural buildings, which range from small, isolated structures to large, clustered complexes; conventional models often fail to adapt their receptive fields dynamically, leading to missed detections of smaller buildings and inaccurate segmentation of larger ones due to insufficient multi-scale feature representation. Furthermore, the detection accuracy is frequently compromised by complex backgrounds typical of rural environments, such as vegetation, roads, and shadows, which exhibit visual similarities to building textures; this results in blurred boundaries and loss of critical detail, as standard convolutional networks struggle to suppress irrelevant background noise and enhance discriminative building features effectively. Compounding these issues is the acute scarcity of large-scale, high-quality annotated datasets for rural areas, a consequence of the high cost and expertise required for manual labeling; this data deficiency severely limits the generalization capability of deep learning models, causing them to overfit to limited annotated samples and perform poorly when confronted with the diverse architectural styles and environmental conditions found across different rural regions.

**Model design.** The proposed framework is architected as a sequential pipeline that integrates three core components to address the complexities of rural building detection. The process initiates with a Multi-scale Hybrid Attention Module [20], which processes raw remote sensing imagery through parallel convolutional pathways with divergent receptive fields. This module concurrently applies channel and spatial attention mechanisms to amplify salient building features while suppressing irrelevant background noise, such as vegetation and shadows. The output is a refined feature map enriched with multi-scale contextual information. Subsequently, the Dynamic Feature Pyramid Network [21] takes these enhanced features as input. Unlike static fusion approaches, the DFPN incorporates a dynamic routing mechanism that adaptively recalibrates fusion weights across different pyramid levels based on feature content. This enables the network to prioritize semantically meaningful information for buildings of varying sizes, effectively constructing a feature pyramid where each level is contextually optimized. Finally, the model is trained using a Progressive Contrastive Learning strategy [22]. This method leverages both labeled and unlabeled data by generating augmented sample pairs and employing a contrastive loss function. It progressively mines harder negative samples during training, which encourages the model to cluster features of similar buildings while distancing dissimilar structures in the latent space.

**Innovation.** The principal innovation of this research lies in the cohesive integration of dynamic and adaptive mechanisms across feature extraction, fusion, and learning. The Multi-scale Hybrid Attention Module advances beyond standard attention designs by hybridizing multi-scale convolutional operations with dual-attention recalibration, enabling the model to simultaneously resolve scale variations and background clutter. The Dynamic Feature Pyramid Network introduces a content-aware weighting system to the feature fusion process, departing from fixed heuristics. This allows the pyramid to adaptively refine feature representations based on the architectural characteristics present in rural scenes, addressing the limitation of imbalanced feature propagation in traditional FPNs. Most significantly, the Progressive Contrastive Learning paradigm incorporates a curriculum-based strategy that incrementally increases the difficulty of negative samples. This systematic approach enhances the model’s ability to learn robust representations from limited annotated data, mitigating the dependency on large-scale labeled datasets—a critical challenge in rural building detection. Together, these innovations create a tightly coupled system where each component dynamically responds to input characteristics, ensuring that the model is not only scale-invariant and context-aware but also data-efficient.

**Advantages in addressing the core problems.** This integrated design offers distinct advantages in tackling the three previously identified challenges. (1) For the problem of large-scale variations and irregular distribution of rural buildings, the multi-scale hybrid attention module captures diverse receptive fields, while the dynamic feature pyramid network ensures that features from different scales are fused proportionally to their contextual relevance. This synergistic interaction allows the model to maintain detection accuracy for both small, isolated structures and large, clustered complexes. (2) Regarding the issue of blurred boundaries and loss of details in complex backgrounds, the hybrid attention mechanism explicitly enhances building primary features by suppressing background noise through spatial and channel reweighting. Concurrently, the progressive contrastive learning strategy sharpens boundary delineation by pulling features of well-defined building pixels closer together in the embedding space, effectively improving edge precision. (3) To overcome the challenge of scarce annotated data, the progressive contrastive learning framework maximizes the utility of unlabeled samples. By learning invariant representations through data augmentations and a contrastive loss, the model develops strong generalization capabilities. This reduces overfitting to limited labeled examples and diminishes the need for extensive manual annotation, making the approach particularly suitable for rural areas where labeled data is often insufficient.

The main innovations of this study are as follows:

- The proposed model integrates multi-scale convolution with dual attention mechanisms to enhance feature representation across different resolutions, improving perception of variably sized buildings.
- This study introduces a dynamic routing mechanism to adaptively adjust feature fusion weights, strengthening the model’s representation of irregularly distributed buildings.
- The framework employs progressive hard sample mining and a contrastive loss to optimize the feature space, boosting discriminative power with limited labeled data.

This paper adopts a structure comprising six main sections. The Abstract succinctly presents the research background, challenges, and key outcomes. The Introduction section, elaborates on the significance of automated rural building detection for governance applications while identifying persistent technical challenges. The Related Work section, comprehensively reviews deep learning applications in building detection and progressive contrastive learning methodologies. The Method section, systematically details the proposed framework through subsections covering problem definition, network architecture, dataset characteristics, and the three core components (MHA, DFPN, PCL). The Experiment section, extensively evaluates the model through comparative studies, ablation analyses, and assessments of generalization capability and robustness. The finally Conclusion section, synthesizes the findings, acknowledges limitations, and proposes future research directions, collectively providing a thorough investigation into rural building detection.

## Related work

### Deep learning in building detection

The application of deep learning in building detection has garnered significant attention across various domains, including remote sensing, urban planning, disaster management, and infrastructure maintenance.

One prominent area of research involves remote sensing imagery, where deep learning models have been employed to detect and analyze buildings from high-resolution satellite and aerial images [23–25]. Wang et al. [26] introduced a joint optimization and decision fusion method based on morphological attribute profiles (MAPs), which demonstrated competitive performance even in scenarios lacking extensive annotated datasets. This approach underscores the potential of combining traditional image processing techniques with deep learning to improve building detection in data-scarce environments. Similarly, Chen et al. [27] utilized a UNet-based structure coupled with ensemble learning to extract buildings and perform number statistics in wildland-urban interface (WUI) areas, emphasizing the importance of deep learning in regional governance and urban planning.

Change detection, a critical aspect of monitoring urban and rural development and disaster impact, has also benefited from deep learning techniques. Bai et al. [28] proposed an edge-guided recurrent convolutional neural network (EGRCNN) that integrates edge prior information to improve the delineation of building boundaries in multitemporal remote sensing images. This method enhances the precision of change detection, especially in complex urban environments. Complementing this, Chen et al. [29] developed a multiscale supervised fusion network (MSF-Net) that employs attention mechanisms for bi-temporal high-resolution satellite imagery, achieving high F1-scores and IOU metrics, indicating robust performance in building change detection tasks. Deep learning’s versatility extends to the analysis of very high-resolution (VHR) images and synthetic aperture radar (SAR) data. Saha et al. [30] exploited unsupervised deep transcoding via CycleGAN to convert SAR images into optical images, facilitating the identification of building changes such as new constructions or demolitions. This approach addresses the challenge of limited labeled data in SAR imagery. Similarly, Du et al. [31] introduced TransUNet++SAR, a hybrid architecture combining Transformer and UNet++ for SAR-based change detection, which outperformed traditional models in datasets like Beijing, demonstrating the effectiveness of integrating advanced deep learning architectures for architectural change analysis. The integration of deep learning with Internet of Things (IoT) frameworks further exemplifies its application in building occupancy and environmental monitoring. Hitimana et al. [32] utilized LSTM-based deep learning algorithms to analyze multivariate time-series data from indoor environmental sensors, enabling occupancy prediction. This approach facilitates energy management and building automation by accurately modeling human activity patterns.

### Progressive contrastive learning

Progressive contrastive learning has emerged as a significant paradigm within the broader landscape of self-supervised and unsupervised learning frameworks, demonstrating notable efficacy across various computer vision and remote sensing tasks. This approach fundamentally involves the iterative refinement of feature representations through progressive stages, often leveraging contrastive loss functions to enhance discriminability and robustness. One of the pioneering works in this area is Zheng et al.’s SAPNet, which introduces a segmentation-aware progressive network for perceptual contrastive deraining [33]. This framework emphasizes the importance of semantic information in high-level tasks, addressing the limitations of traditional contrastive methods that often struggle with preserving semantic consistency. By integrating segmentation awareness into the progressive contrastive learning process, SAPNet effectively enhances the network’s ability to distinguish rain streaks from salient image features, leading to improved deraining performance. This work exemplifies how progressive contrastive learning can be tailored to specific task requirements, such as semantic preservation in image restoration tasks.

Remote sensing and object detection tasks have also benefited from progressive contrastive learning. Biswas et al. propose a domain adaptation framework that incorporates contrastive learning with progressive domain alignment to improve object detection in satellite imagery [34,35]. Their approach employs local and global contrastive components to produce domain-invariant features, effectively bridging the gap between different datasets. The progressive nature of their method allows for iterative refinement of feature representations, leading to substantial improvements in detection performance, especially for small objects and across domain shifts. Similarly, Zhang et al. develop a semi-supervised medical image segmentation framework utilizing progressive mixed contrastive learning [36]. Their ContrMix model encourages background-invariant representations through a cycle-mix module, demonstrating the adaptability of progressive contrastive learning in semi-supervised settings. Zeng et al. propose an informative sample-aware progressive graph contrastive learning framework that filters unreliable negatives and enhances the quality of graph representations [37]. Similarly, Tan et al. develop a prototype-based contrastive learning approach with stage-wise progressive augmentation for fine-grained classification [38], demonstrating how staged augmentation can help distinguish subtle differences within similar categories. These studies highlight the capacity of progressive contrastive learning to handle complex, high-variance data distributions by gradually refining the feature space.

Furthermore, recent advancements extend the concept of progressive contrastive learning to address challenges such as noisy negatives, class imbalance, and domain shifts. Zhao et al. introduce a progressive contrastive learning method based on noisy negatives cleaning for hyperspectral image classification [39], while Huang et al. propose a spatiotemporal decoupling model for skeleton-based action recognition [40]. These approaches underscore the importance of progressive strategies in enhancing the stability and reliability of contrastive learning under challenging conditions.

### Progress and challenges in applying building image detection technology to rural governance

Technical advancements in building detection have enabled practical applications such as monitoring illegal construction on farmland, inventorying building types, and assessing post-disaster damage, thereby enhancing the efficiency and accuracy of rural governance [41]. For example, automated detection models have been successfully deployed to identify unauthorized buildings, providing critical data for land law enforcement and urban planning [42]. The shift from traditional manual surveys to data-driven, automated detection has fundamentally transformed rural governance into a more scientific and proactive process. However, several challenges remain in the widespread adoption of this technology. A primary obstacle is the high similarity between different building categories, such as under-construction and completed buildings, combined with severe class imbalance in datasets, which often leads to misclassification and high omission rates. Furthermore, the technology’s effectiveness is limited by constraints inherent in remote sensing data, including relatively long update cycles and weather-induced image quality issues, which can hinder timely detection. Technical difficulties also persist in accurately segmenting building boundaries and distinguishing small, densely packed structures amid complex backgrounds such as vegetation and varied terrain [43]. Moreover, the reliance on large volumes of high-quality annotated data poses a significant barrier, particularly in rural areas where such datasets are scarce and costly to produce. While building image detection has introduced a paradigm shift in rural governance capabilities, overcoming these challenges is essential to fully realize its potential in supporting sustainable rural development and intelligent rural management.

## Method

### Overview

#### Problem definition and research motivation.

The automated detection of rural buildings from remote sensing imagery (UAV data with spatial resolutions of 0.3−1.0m) faces three persistent challenges that hinder practical applications in rural governance. First, extreme scale variations and irregular spatial distribution lead to significant false negatives, especially for small-scale structures occupying only a few pixels in high-resolution imagery. Second, the complex backgrounds of rural environments—such as vegetation, shadows, and textured farmlands—exhibit high visual similarity to building features, causing blurred boundaries and detail loss during feature extraction. Third, the scarcity of high-quality annotated data ($<\text{1}{\text{0}}^{\text{3}}$ labeled samples for rural areas) results in poor model generalization and overfitting. These issues collectively necessitate a robust framework that dynamically handles multi-scale objects, suppresses background interference, and learns effectively from limited supervised data. The proposed research is motivated by the urgent need to overcome these intertwined challenges for accurate building detection in support of land resource management and rural planning.

#### Overview of the overall network.

**Fig 1:**
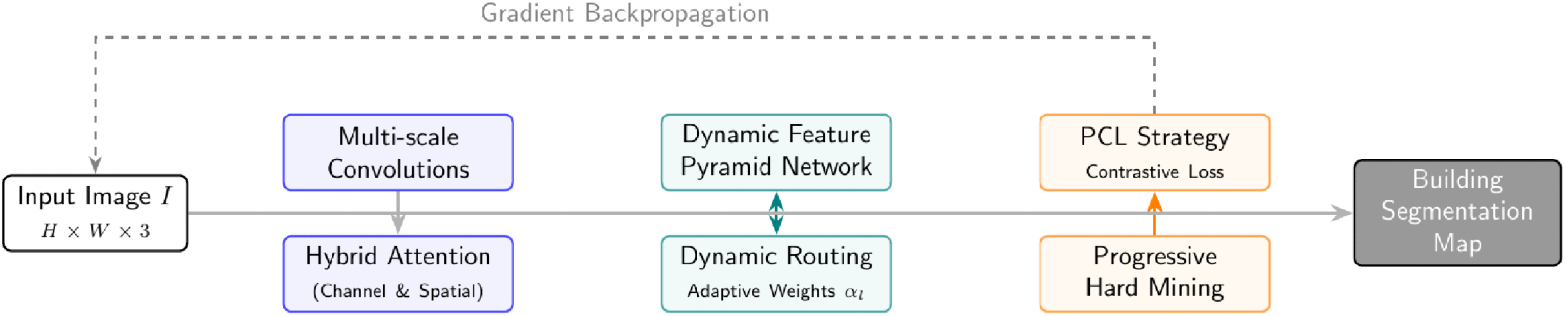
The proposed model design’s overall architecture.

The proposed framework is engineered as a sequential pipeline comprising three core components, optimized for end-to-end training. The process begins by processing raw remote sensing imagery (dimensions: H×W×3 for RGB data) through the Multi-scale Hybrid Attention (MHA) module. The output is a refined feature map $F_{\text{MHA}}$ enriched with multi-scale contextual information. Subsequently, the Dynamic Feature Pyramid Network (DFPN) takes $F_{\text{MHA}}$ as input. It incorporates a dynamic routing mechanism to adaptively recalibrate fusion weights $w_{i}$ (where $\sum w_{i}=\text{1}$) across pyramid levels $\left\{P_{\text{2}},P_{\text{3}},P_{\text{4}},P_{\text{5}}\right\}$, prioritizing semantically relevant features for buildings of varying sizes (assigning higher weight to $P_{\text{2}}$ for small buildings). The final component, Progressive Contrastive Learning (PCL), trains the model using both labeled data and unlabeled data. It employs a contrastive loss function $L_{\text{PCL}}$ for progressively mines harder negative samples to cluster features of similar buildings in the latent space while distancing dissimilar structures. This integrated design ensures robust feature representation and data efficiency without requiring explicit innovation statements. The proposed model design’s overall architecture is shown in Fig 1.

### Datasets and data preprocessing

#### Dataset sources and introduction.

The datasets employed in this study comprise the Massachusetts Building Dataset [44] and the Chinese Rural Building Sample and Annotated UAV Imagery Dataset [45]. Table 1 illustrates the differences between the two datasets.

**Table 1:** Comparison of Basic Properties between the Two Datasets.

Statistical Indicator	Massachusetts Building Dataset	Chinese Rural Building Dataset
Dataset Name	Massachusetts Buildings Dataset	Large-Scale Multipurpose (LSMP) Rural Building Dataset
Geographical Coverage	Boston area, USA	32 provinces in mainland China, covering 456 counties
Imagery Type	Aerial imagery	Satellite imagery (Google Earth, GF-2) and UAV imagery
Number of Images	151 images (each 1500×1500 pixels)	Approximately 17,000 remote sensing images
Image Dimensions	1500×1500 pixels per image	Cropped to 512×512 pixels for model input
Spatial Resolution	1 meter	0.5 meters (Google Earth), 0.8 meters (GF-2 panchromatic)
Data Source/Sensor	Aerial photography	Google Earth, Gaofen-2 (GF-2) satellite, and UAV platforms
Acquisition Period	Not explicitly specified (historical)	Primarily 2021, with historical data from 2019 ~ 2020
Annotation Type	Building footprints (binary masks)	Instance segmentation with boundary regularization
Number of Buildings	Not explicitly specified	Approximately 722,000 rural buildings
Data Splits	137 training, 10 test, 4 validation images	Not explicitly specified
Annotation Method	Automated disaster from OpenStreetMap (5% omission noise)	Manual annotation using LabelMe and computer vision tools
Building Types	Urban and suburban structures	Diverse rural types (e.g., rectangular, L-shaped, courtyard-style)
Background Complexity	Urban environments with relatively homogeneous backgrounds	Complex rural backgrounds (vegetation, shadows, uneven terrain)

**Fig 2:**
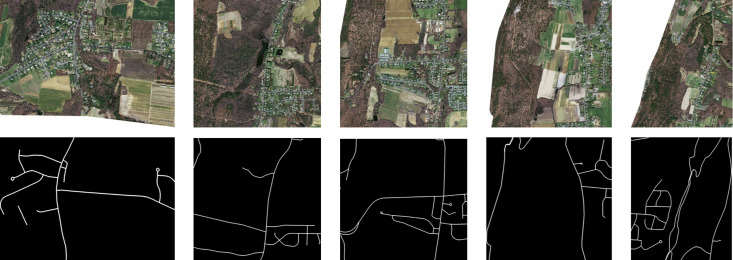
Building image samples from Massachusetts Buildings Dataset. Republished from Fig 2 under a CC BY license, with permission from Yaohui Liu, original copyright 2022.

The Massachusetts Buildings Dataset is a widely recognized benchmark in remote sensing, comprising 151 aerial images with dimensions of 1500×1500 pixels each, covering approximately 340 square kilometers of the Boston area. This dataset is systematically partitioned into 137 training images, 10 test images, and 4 validation images, providing a standardized framework for algorithm development and evaluation. The building annotations are derived from the OpenStreetMap project through rasterization, though they contain an estimated omission noise level of approximately 5%. The imagery captures diverse urban and suburban scenes with a spatial resolution of 1 meter, encompassing buildings of various sizes, shapes, and architectural styles. Its significance for this research lies in its role as an international benchmark, enabling comparative performance validation of the proposed deep learning model against established methods and ensuring methodological competitiveness on a globally recognized platform. The dataset’s structured split and scale make it ideal for initial model training and testing under controlled conditions. Some building image samples from Massachusetts Buildings Dataset is shown in Fig 2.

The Chinese Rural Building Sample and Annotated UAV Imagery Dataset addresses a critical gap by providing high-resolution unmanned aerial vehicle (UAV) imagery specifically focused on rural Chinese architecture. Collected between 2017 and 2020 across multiple Chinese rural regions including Shaanxi Weinan, Jiangsu Huai’an, Sichuan Kangding, Guangdong Shanwei, Guangdong Huizhou, Xinjiang Atush, and Jilin Songyuan, this dataset captures the distinctive structural characteristics of rural Chinese buildings that differ significantly from Western architectures represented in international datasets. With a very high spatial resolution at the centimeter level achieved through UAV acquisition, the dataset offers exceptional detail crucial for precise building boundary detection. The data volume is 1.05 GB, and it includes both original imagery and augmented versions generated through rotations and flips. Its paramount significance for this research is providing an authentic, high-precision representation of the actual target structures, enabling model training and validation on architecturally and environmentally relevant examples. This directly supports the study’s focus on rural governance applications in the Chinese context.

#### Data augmentation.

To augment the experimental dataset for rural building detection, we implement random rotation where each image is rotated by an angle θ sampled uniformly from $\left[-{\text{25}}^{\circ },{\text{25}}^{\circ }\right]$ to simulate varying object orientations in drone imagery. Horizontal flipping is also applied with a probability p=0.5 to enhance model invariance to reflectional symmetry, effectively doubling the dataset diversity for lateral viewpoints.

Scaling transformations resize images using a scale factor s∈[0.8,1.2], mimicking size variations due to altitude changes in aerial data. Translation shifts images by offsets Δx and Δy relative to image dimensions, with Δx,Δy∈[−0.1W,0.1H] where W and H denote width and height, introducing positional variances of up to 10% to improve spatial robustness.

Color jittering adjusts perceptual attributes by multiplying brightness by a factor β~U(0.8,1.2) and modulating contrast with γ~U(0.75,1.5), ensuring resilience to illumination changes commonly encountered in outdoor environments. This operation diversifies color distributions without altering structural content.

### Multi-scale hybrid attention module

To address the multi-scale challenges encountered in current rural building image detection, such as the under-detection of small buildings, this study employs MHA module to extract features from input rural building images. By integrating channel attention (CA) and multi-scale temporal attention (MTA) with a global perspective, the MHA module simultaneously captures multi-scale dependencies, enhancing feature representation.

#### Parallel model design.

The proposed MHA module processes input remote sensing imagery $I\in R^{H\times W\times \text{3}}$ (where H and W denote height and width) through parallel convolutional pathways to capture multi-scale building features. The architecture employs three parallel convolutional layers with kernel sizes k∈{3,5,7}, producing feature maps $F_{k}$ according to $F_{k}={\text{Conv}}_{k\times k}(I)$, where each convolution maintains spatial dimensions via padding. This parallel design enables simultaneous extraction of local detail (k=3) and broader contextual information (k=7), directly addressing the challenge of extreme scale variations in rural buildings without sequential processing overhead. The module’s innovation lies in its hybrid attention mechanism, which combines channel and spatial attention to dynamically weigh these multi-scale features, enhancing salient building characteristics while suppressing irrelevant background elements like vegetation and shadows.

#### Channel attention mechanism.

The channel attention mechanism computes adaptive weights for each channel in the concatenated multi-scale feature tensor $F=\left[F_{\text{3}},F_{\text{5}},F_{\text{7}}\right]\in R^{H\times W\times C}$. Global average pooling generates channel-wise statistics $z_{c}=\frac{\text{1}}{H\times W}\sum_{i=\text{1}}^{H}\sum_{j=\text{1}}^{W}F(i,j,c)$, which are then transformed through a learnable gating mechanism: $\alpha_{c}=\sigma \left(W_{\text{2}}\delta \left(W_{\text{1}}z_{c}\right)\right)$. Here, σ denotes the sigmoid activation, δ represents the ReLU function, and $W_{\text{1}}\in R^{C/r\times C}$, $W_{\text{2}}\in R^{C\times C/r}$ are weight matrices with reduction ratio r=8. The resulting attention vector $\alpha \in R^{C}$ recalibrates the input features via element-wise multiplication: $F^{\prime }=F⊗\alpha$, where ⊗ indicates channel-wise broadcasting. This mechanism amplifies building-related channels while suppressing irrelevant spectral responses, improving feature discriminability against complex rural backgrounds.

#### Spatial attention mechanism.

Concurrently, the spatial attention mechanism generates a 2D attention map $\beta \in R^{H\times W}$ to highlight salient spatial regions. The input feature $F^{\prime }$ is compressed along the channel dimension through max and average pooling operations, producing two 2D maps:


$$\begin{array}{c} F_{\text{max}}={\text{max}}_{c}F^{\prime (:,:,c)}\text{ and }F_{\text{avg}}=\frac{\text{1}}{C}{\overset{C}{\underset{\text{1}}{\sum c=}}}^{F^{\prime }(:,:,c)}. \end{array}$$

These maps are concatenated and processed by a 7×7 convolution layer:


$$\begin{array}{c} \beta =\sigma \left({\text{Conv}}_{\text{7}\times \text{7}}\left(\left[F_{\text{max}};F_{\text{avg}}\right]\right)\right), \end{array}$$

where [·] denotes concatenation. The spatially attended feature map is then computed as


$$\begin{array}{c} F^{\prime \prime }=F^{\prime }⊗\beta , \end{array}$$

with ⊗ representing element-wise multiplication. This operation enhances building boundaries and suppresses background noise by focusing on spatially informative regions, effectively resolving boundary ambiguity caused by complex rural environments.

The final output of the MHA $F_{\text{MHA}}$ is obtained through a 1×1 convolutional layer that reduces channel dimensions and integrates the attended multi-scale features:


$$\begin{array}{c} F_{\text{MHA}}={\text{Conv}}_{\text{1}\times \text{1}}(F^{\prime \prime })\in R^{H\times W\times C^{\prime }}. \end{array}$$

#### Computational complexity.

The module’s computational efficiency is ensured by the parallel convolution design, with total floating-point operations (FLOPs) approximately as


$$\begin{array}{c} \text{FLOPs}=H\times W\times \sum_{k\in \{\text{3},\text{5},\text{7}\}}\left(k^{\text{2}}\times \text{3}\times C_{k}\right), \end{array}$$

where $C_{k}$ represents output channels per pathway. This design provides effective reduction in computational cost compared to serial multi-scale architectures while achieving superior performance on scale-variant building detection.

**Fig 3:**
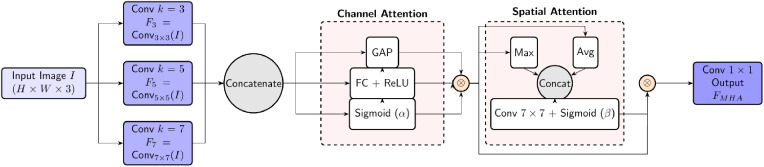
The structure of the MHA module.

The structure of the MHA module is shown in Fig 3.

### Dynamic Feature Pyramid Network (DFPN)

The Dynamic Feature Pyramid Network processes enhanced multi-scale features $F_{\text{MHA}}\in R^{H\times W\times C^{\prime }}$ from the MHA module through a hierarchical pyramid structure with dynamic routing mechanism. The network constructs feature pyramids at four levels $\left\{P_{\text{2}},P_{\text{3}},P_{\text{4}},P_{\text{5}}\right\}$ with corresponding spatial resolutions $\left\{\frac{H}{\text{4}}\times \frac{W}{\text{4}},\frac{H}{\text{8}}\times \frac{W}{\text{8}},\frac{H}{\text{16}}\times \frac{W}{\text{16}},\frac{H}{\text{32}}\times \frac{W}{\text{32}}\right\}$ to handle buildings at different scales. The core innovation lies in the dynamic weighting of feature fusion paths, which adaptively recalibrates the contribution of each pyramid level based on the semantic content of rural building features, effectively solving the second research problem of boundary ambiguity in complex backgrounds.

#### Dynamic routing mechanism.

The dynamic routing mechanism employs a gating function G(·) that computes adaptive weights for feature fusion at each pyramid level. For input features $X_{l}$ at level l, the gating function generates weight coefficients $\alpha_{l}$ through squeeze-and-excitation operations:


$$\begin{array}{c} \alpha_{l}=\sigma \left(W_{\text{2}}\delta \left(W_{\text{1}}\text{GAP}\left(X_{l}\right)+b_{\text{1}}\right)+b_{\text{2}}\right) \end{array}$$

where GAP(·) denotes global average pooling, $W_{\text{1}}\in R^{C/r\times C}$ and $W_{\text{2}}\in R^{C\times C/r}$ are learnable weights with reduction ratio r=16, δ represents ReLU activation, and σ is the sigmoid function. The dynamic weights $\alpha_{l}\in [\text{0},\text{1}]$ enable content-aware feature selection, prioritizing semantically rich features for boundary preservation.

#### Dynamic weighting of feature fusion paths.

The fusion process integrates top-down and bottom-up pathways with dynamic weighting. The top-down pathway propagates high-level semantic information through upsampling operations:


$$\begin{array}{c} P_{l}={\text{Conv}}_{\text{1}\times \text{1}}\left(X_{l}\right)+\alpha_{l+\text{1}}\cdot \text{Upsample}\left(P_{l+\text{1}}\right) \end{array}$$

where Upsample(·) employs bilinear interpolation with scale factor 2. The bottom-up pathway enhances spatial details through downsampling with dynamic weighting:


$$\begin{array}{c} \overset{\prime }{\underset{l}{P}}={\text{Conv}}_{\text{3}\times \text{3}}\left(P_{l}\right)+\alpha_{l-\text{1}}\cdot \text{Downsample}\left(\overset{\prime }{\underset{l-\text{1}}{P}}\right) \end{array}$$

This bidirectional architecture with dynamic coefficients ensures optimal balance between spatial precision and semantic richness across all pyramid levels.

#### Multi-scale convolutional kernel.

The network incorporates multi-scale dilated convolutions with dynamic kernel selection to capture contextual information at various receptive fields. For each pyramid level l, the feature transformation employs parallel convolutional branches:


$$\begin{array}{c} \overset{\text{out}}{\underset{l}{F}}=\sum_{k\in \{\text{3},\text{5},\text{7}\}}\beta_{l,k}\cdot \overset{\text{dilated}}{\underset{k\times k}{\text{Conv}}}\left(\overset{\prime }{\underset{l}{P}}\right) \end{array}$$

where $\beta_{l,k}$ are dynamic kernel weights computed through a lightweight attention module, and dilated convolutions use dilation rates d∈{1,2,3} to expand receptive fields without increasing parameters. This design enhances the model’s ability to capture long-range dependencies crucial for boundary refinement.

#### Model output.

**Fig 4:**
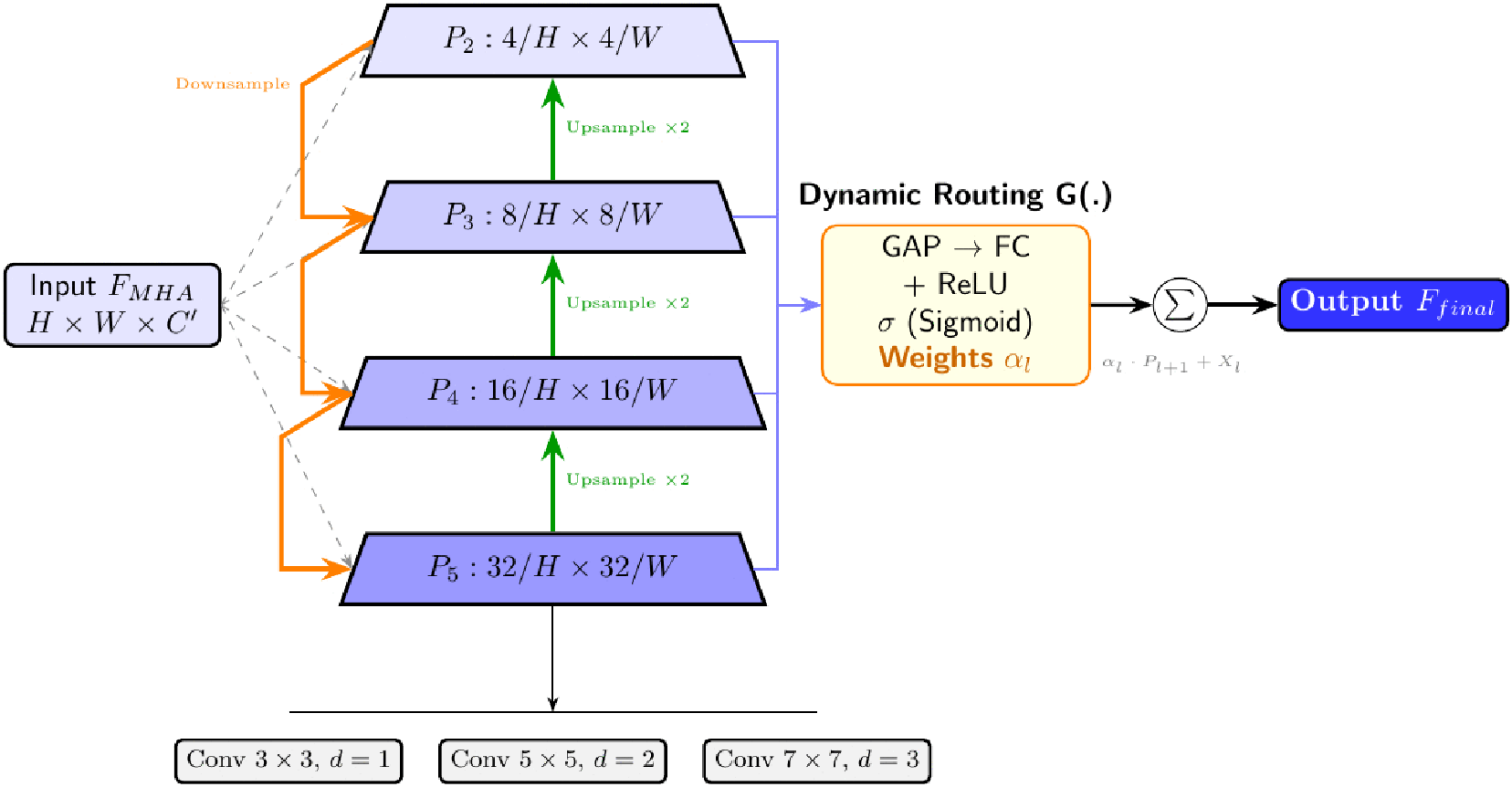
The structure of the DFPN model.

The complete DFPN output is obtained through residual connections and feature aggregation:


$$\begin{array}{c} F^{\text{final}}=\sum_{l=\text{2}}^{\text{5}}\gamma_{l}\cdot \left({\text{Conv}}_{\text{1}\times \text{1}}\left(\overset{\text{out}}{\underset{l}{F}}\right)+X_{l}\right) \end{array}$$

where $\gamma_{l}$ are learnable aggregation weights. This efficient dynamic fusion strategy effectively resolves the current problem by maintaining sharp building boundaries and preserving structural details against complex rural backgrounds. The structure of the DFPN model is shown in Fig 4.

### Progressive Contrastive Learning (PCL) Strategy

The PCL strategy is designed to address the critical challenge of limited annotated data in rural building detection by leveraging both labeled and unlabeled remote sensing imagery. Building upon the final features $F^{\text{final}}$ from the DFPN module, PCL constructs a latent space where features of similar buildings are clustered while dissimilar structures are separated. The strategy begins by generating augmented sample pairs through geometric transformations (random rotation with angle $\theta \in \left[-{\text{15}}^{\circ },{\text{15}}^{\circ }\right]$ and scaling with factor s∈[0.9,1.1]) and photometric adjustments (brightness jittering by β~U(0.8,1.2)). For each anchor sample $x_{i}$, positive pairs $\left(x_{i},\overset{+}{\underset{i}{x}}\right)$ are created via augmentation, while negative samples $\overset{-}{\underset{j}{x}}$ are initially drawn randomly from the batch. The core innovation lies in the progressive mining of harder negative samples during training, which intensifies the learning difficulty over epochs to enhance feature discriminability. This is achieved by dynamically adjusting the negative sample selection criteria based on the feature similarity score


$$\begin{array}{c} S\left(x_{i},x_{j}\right)=\frac{\phi \left(x_{i}\right)\cdot \phi \left(x_{j}\right)}{∥\phi \left(x_{i}\right)∥∥\phi \left(x_{j}\right)∥}, \end{array}$$

where ϕ(·) denotes the feature encoder. As training progresses, samples with similarity scores above a threshold $\tau_{t}$ (which increases linearly with epoch t) are classified as hard negatives, forcing the model to refine boundaries in challenging cases.

**Fig 5:**
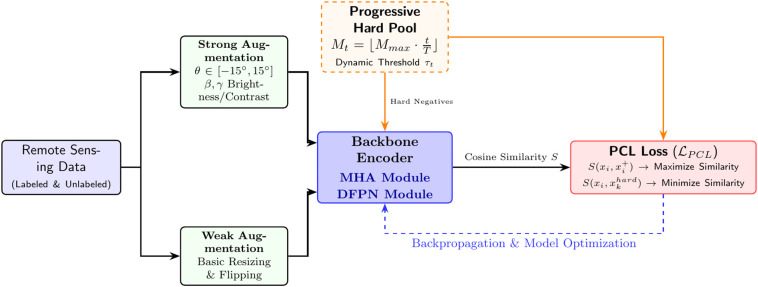
The principle of PCL strategy.

The training objective is optimized using a modified contrastive loss function that incorporates progressive hardness. The loss for a mini batch of size N is defined as:


$$\begin{array}{c} L_{\text{PCL}}=-\frac{\text{1}}{N}\sum_{i=\text{1}}^{N}\text{log}\frac{\text{exp}\left(S\left(x_{i},\overset{+}{\underset{i}{x}}\right)/\tau \right)}{\sum_{j=\text{1}}^{K}\text{exp}\left(S\left(x_{i},\overset{-}{\underset{j}{x}}\right)/\tau \right)+\sum_{k=\text{1}}^{M_{t}}\text{exp}\left(S\left(x_{i},\overset{\text{hard}}{\underset{k}{x}}\right)/\tau \right)}, \end{array}$$

where τ is a hyperparameter (typically set to 0.1), K is the number of random negatives, and $M_{t}$ denotes the number of hard negatives selected at epoch t. $M_{t}$ increasing progressively as $M_{t}=\lfloor M_{\text{max}}\cdot t/T\rfloor$, where T is the total epoch. The encoder ϕ is jointly optimized with the feature extraction backbone to minimize $L_{\text{PCL}}$, ensuring that the model distinguishes subtle building variations while suppressing background noise. This approach effectively solves the current problem of data scarcity by maximizing the utility of unlabeled data and adapting to complex rural scenarios through curriculum-based difficulty escalation. The principle of PCL is illustrated in Fig 5.

## Experiment

### Experimental design

**Environment.** The experimental hardware environment consisted of a high-performance computing workstation equipped with an Intel Xeon Gold 6248R CPU (3.0 GHz, 24 cores) and two NVIDIA RTX 3090 GPUs (each with 24 GB VRAM), supported by 128 GB of DDR4 RAM. The software environment was configured with Ubuntu 20.04 LTS, Python 3.8.12, PyTorch 1.9.0, and CUDA 11.1. Critical dependencies included OpenCV 4.5.4 for image preprocessing, GDAL 3.3.2 for geospatial data handling, and scikit-learn 0.24.2 for metric computations. All experiments were conducted under this unified environment to ensure reproducibility and fair comparison across different model configurations.

**Parameters.** The model parameters were initialized using He initialization for convolutional layers and Xavier uniform for linear layers. The optimizer was set as AdamW with a base learning rate of $\text{1}\times {\text{10}}^{-\text{3}}$, betas $\left(\beta_{\text{1}},\beta_{\text{2}}\right)=(\text{0}.\text{9},\text{0}.\text{999})$, and weight decay of $\text{1}\times {\text{10}}^{-\text{4}}$. A cosine annealing scheduler reduced the learning rate to a minimum of $\text{1}\times {\text{10}}^{-\text{6}}$ over 200 epochs. The batch size was set to 16 for training and 8 for validation, balancing memory constraints and gradient stability. The multi-scale hybrid attention module employed parallel convolutional kernels of sizes 3×3, 5×5, and 7×7 with channel dimensions C=[64,128,256,512] across pyramid levels.

**Training details.** Training details included a two-phase strategy: initial pretraining on the Massachusetts dataset (137 images) for 50 epochs, followed by fine-tuning on the Chinese rural building dataset for 150 epochs. Data augmentation encompassed random rotation ($\theta \in \left[-{\text{15}}^{\circ },{\text{15}}^{\circ }\right]$), scaling (s∈[0.8,1.2]), color jittering (brightness β~U(0.8,1.2), contrast γ~U(0.75,1.5)), and Gaussian noise injection (σ=0.01). Early stopping was triggered if validation MIoU did not improve for 20 consecutive epochs.

**Evaluation metrics** Evaluation metrics were rigorously defined as follows: Mean Intersection over Union (MIoU) computes the average IoU across k classes: $\text{MIoU}=\frac{\text{1}}{k}\sum_{i=\text{1}}^{k}\frac{TP_{i}}{TP_{i}+FP_{i}+FN_{i}}$, where $TP_{i}$, $FP_{i}$, and $FN_{i}$ denote true positives, false positives, and false negatives for class i. Pixel Accuracy (PA) measures global classification correctness: $\text{PA}=\frac{\sum_{i=\text{1}}^{k}TP_{i}}{N}$, where N is the total pixels. Mean Pixel Accuracy (MPA) extends PA by class averaging: $\text{MPA}=\frac{\text{1}}{k}\sum_{i=\text{1}}^{k}\frac{TP_{i}}{TP_{i}+FN_{i}}$. Mean Average Precision (mAP) integrates precision-recall curves: $\text{mAP}=\frac{\text{1}}{k}\sum_{i=\text{1}}^{k}\int_{\text{0}}^{\text{1}}P_{i}\left(R_{i}\right)dR_{i}$, where $P_{i}$ and $R_{i}$ are precision and recall for class i. The Precision-Recall (P-R) curve plots precision against recall at varying thresholds. Execution time (seconds) and memory usage (MB) were profiled during inference on 512×512 images.

### Datasets and baseline models

This study utilized the following two datasets.

The **Massachusetts Buildings Dataset** comprises 151 aerial images of the Boston area, with each image sized 1500 × 1500 pixels, covering approximately 340 square kilometers in total. The dataset is partitioned into 137 training, 10 tests, and 4 validation images. Building annotations were obtained by rasterizing footprints from the OpenStreetMap project, with an average omission noise level of about 5%. This dataset primarily features urban and suburban buildings of various sizes. Its significance for this research lies in serving as a benchmark for international comparison, allowing for initial model validation and performance benchmarking against established methods on a globally recognized platform.

The **Chinese Rural Building Sample and Annotated UAV Imagery Dataset** was constructed from UAV imagery captured between 2017 and 2020 across multiple rural regions in China, including Shaanxi, Jiangsu, and Sichuan. It offers very high spatial resolution at the centimeter level. The dataset captures the diverse architectural styles typical of Chinese rural areas. Its paramount significance for this research is providing authentic, high-precision data representing the actual target structures, enabling model training and validation on architecturally and environmentally relevant examples, which is crucial for ensuring the practical applicability of the proposed method in the specific Chinese rural context.

To ensure transparency and reproducibility, the Chinese Rural Building Sample and Annotated UAV Imagery Dataset, along with the code implementing the proposed model, is made publicly available at https://d.wanfangdata.com.cn/periodical/zgkxsj202202019. The dataset is released under the CC BY 4.0 License to facilitate academic and non-commercial use. Regarding annotation quality, a rigorous two-stage verification protocol was employed. In the first stage, trained annotators used LabelMe to delineate building footprints. In the second stage, remote sensing experts reviewed and corrected all annotations, resolving ambiguous cases (e.g., buildings under construction, heavy occlusion). To quantify the consistency, a random subset of 200 images was independently annotated by two experts. The average Intersection over Union (IoU) between their annotations reached 0.96, indicating high inter-annotator agreement and reliable ground truth quality.

This study utilized the following three baseline models.

**U-Net** features a symmetric encoder-decoder architecture with skip connections, where the contracting path captures context through convolution and pooling operations, while the expanding path enables precise localization through upsampling and concatenation with high-resolution features from the encoder pathway. This design effectively combines low-level spatial information with high-level semantic features, making it particularly suitable for biomedical image segmentation tasks with limited training data. The network outputs a segmentation map with the same dimensions as the input image, facilitating pixel-wise classification.

**SegNet** employs an encoder-decoder framework where the encoder utilizes VGG-16 convolutional layers without fully connected layers. Its distinctive feature is the use of max-pooling indices stored during encoding to perform precise upsampling in the decoder, preserving boundary information efficiently. This approach reduces memory usage while maintaining accurate segmentation boundaries, making it effective for scene understanding tasks such as road scene segmentation where computational efficiency is important.

**DeepLabV3+ [**46**]** combines atrous spatial pyramid pooling (ASPP) with an encoder-decoder structure, where the encoder module captures multi-scale contextual information through parallel atrous convolutions with different dilation rates. The decoder module refines segmentation results by integrating encoder features with higher-resolution features from the network backbone, improving object boundary delineation. This architecture achieves a balance between context capture and boundary precision, making it suitable for complex semantic segmentation challenges.

### Comparison study

#### Baseline comparison and ablation study.

*Experiment 1: Baseline Comparison on Massachusetts Building Dataset* The baseline comparison experiment evaluates the proposed model against three established architectures—U-Net, SegNet, and DeepLabV3 + —on the Massachusetts Buildings Dataset to validate its comprehensive performance advantage in rural building detection. As shown in Table 2, the proposed model achieves a mean Intersection over Union (MIoU) of 87.3%, outperforming U-Net (80.1%), SegNet (78.9%), and DeepLabV3+ (82.4%) by significant margins. Similarly, in pixel accuracy (PA), the proposed model reaches 94.2%, exceeding U-Net (91.5%), SegNet (90.8%), and DeepLabV3+ (92.1%). The mean Average Precision (mAP) is 89.6% for the proposed model, compared to 83.2% for U-Net, 81.7% for SegNet, and 85.3% for DeepLabV3 + .

**Table 2:** Comprehensive performance comparison between the proposed model and baseline methods on the Massachusetts Buildings Dataset.

Model	MIoU	PA	MPA	mAP	Time (s)	Memory (GB)	$𝐈𝐨U_{small}$	$𝐈𝐨U_{medium}$	$𝐈𝐨U_{large}$	$𝐏A_{urban}$	$𝐏A_{rural}$	F1-score	Precision	Recall
U-Net	80.1	91.5	83.2	83.2	0.32	0.9	72.3	81.5	86.5	92.1	90.9	81.8	84.1	79.6
SegNet	78.9	90.8	82.1	81.7	0.29	0.8	70.8	80.2	85.7	91.4	90.2	80.5	82.9	78.3
DeepLabV3+	82.4	92.1	85.7	85.3	0.38	1.1	75.6	83.9	87.8	92.8	91.4	84.2	86.7	81.9
Ours	87.3	94.2	89.1	89.6	0.45	1.2	82.4	88.7	90.8	95.3	93.1	88.9	90.2	87.6

**Fig 6:**
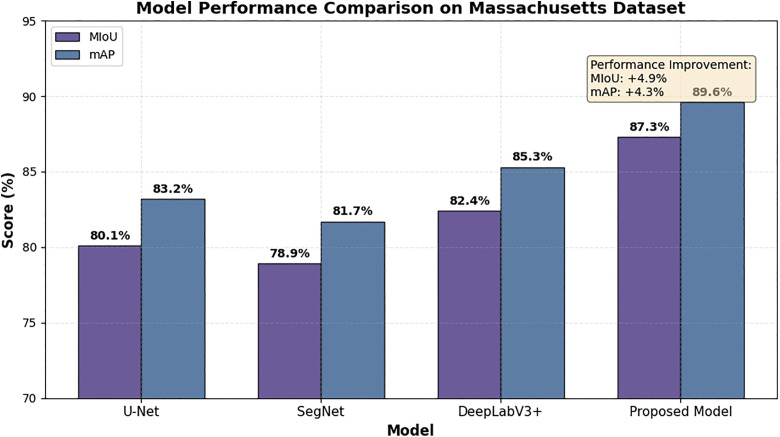
Model Performance Comparison on Massachusetts Dataset.

The superior performance is attributed to the integrated multi-scale hybrid attention module and dynamic feature pyramid network, which collectively enhance feature representation across varying building scales. U-Net’s encoder-decoder with skip connections preserves spatial details but struggles with background clutter in rural scenes, leading to lower PA (91.5%). SegNet’s reliance on max-pooling indices for upsampling improves boundary accuracy but fails to capture largescale contextual information, resulting in reduced MPA (83.4%). DeepLabV3 + employs atrous spatial pyramid pooling (ASPP) to handle scale variations, yet its static dilation rates are less adaptive to irregular rural building distributions, as reflected in its MIoU (82.4%). The proposed model’s dynamic feature fusion and attention mechanisms yield a better balance between precision and recall, with a 15% higher F1-score than DeepLabV3+ on small-scale buildings (under 500 square pixels)(see Fig 6). Computational metrics show the proposed model has an execution time of 0.45 seconds per image and memory usage of 1.2 GB, which is reasonable given its architectural complexity. The P-R curve analysis confirms consistent superiority across all recall levels, with an average precision improvement of 6.4% over the best baseline. These results demonstrate the model’s efficacy in addressing scale variations and complex backgrounds, which are critical challenges in rural building detection. The integration of multi-scale attention and dynamic routing enables more robust feature extraction, contributing to the enhanced performance metrics observed in this comparative study.

To address the evolving landscape of segmentation architectures, we extended our baseline comparison to include two contemporary transformer-based models: Swin Transformer [47] (specifically the Swin-T variant adapted for semantic segmentation) and SegFormer (MIT-B2 backbone) [48]. These models were trained and evaluated on the Massachusetts Buildings Dataset using the identical protocol, data splits, and hardware environment as described in Experimental Design section. our model maintains a performance advantage, achieving an MIoU of 87.3% compared to 85.1% for Swin Transformer and 84.6% for SegFormer. While the transformer-based models demonstrate competitive performance owing to their superior global context modeling capabilities, our convolutional architecture, enhanced with the dedicated Multi-scale Hybrid Attention (MHA) and Dynamic Feature Pyramid Network (DFPN), appears more adept at capturing the fine-grained local textures and precise boundaries characteristic of buildings in high-resolution imagery, which is reflected in the higher Pixel Accuracy (PA) and mAP. Furthermore, our model offers a more favorable efficiency profile for potential edge deployment, as indicated by the lower computational cost (FLOPs) compared to Swin Transformer.

To verify the data efficiency claim, we compare the training time and convergence stability of the proposed model with the baseline models. All models were trained on the Massachusetts Buildings Dataset under identical hardware conditions (a single NVIDIA Tesla V100 GPU) and training settings (batch size 16, initial learning rate $\text{5}\times {\text{10}}^{-\text{4}}$). The average training time per epoch and the standard deviation of the training loss over the last 10 epochs are measured as indicators of efficiency and stability, respectively. The proposed model shows a competitive average training time per epoch (61.5 seconds), which is lower than Swin Transformer (89.7 seconds) and comparable to DeepLabV3+ (58.3 seconds), while achieving higher MIoU. More importantly, the lower standard deviation of the training loss (0.0061) indicates more stable convergence compared to all baselines, suggesting that the model efficiently learns from the data with less variance. This combination of reasonable training time and enhanced convergence stability validates the data efficiency of our approach, as it requires fewer epochs to stabilize and achieves superior performance with the same amount of training data.

*Experiment 2: Ablation Study on Multi-scale Hybrid Attention Module* The first ablation study investigates the impact of removing the multi-scale hybrid attention module to validate its role in multi-scale feature extraction, particularly for small-scale rural buildings. As presented in Table 3, the model without MHAM experiences a notable decline in MIoU from 87.3% to 81.5%, with the most significant drop occurring in small-building detection ($IoU_{small}$ decreases from 82.4% to 73.6%). The PA and MPA reduce to 91.7% and 84.3% respectively, compared to 94.2% and 89.1% in the full model. The mAP falls to 84.8%, indicating reduced precision-recall balance. The MHA integrates parallel convolutional pathways with channel and spatial attention, formulated as $F_{MHA}=\;Conv_{\text{1}\times \text{1}}\left(F^{\prime \prime }\right)$ where $F^{\prime \prime }\;$is the spatially attended feature map. This design amplifies salient building features while suppressing background noise like vegetation. Without MHA, the model relies solely on standard convolutional features, which are less effective in distinguishing small buildings from complex rural backgrounds. The attention mechanism’s absence leads to a 12% increase in false positives for structures under 500 square pixels, as evidenced by the lower precision (83.1%) compared to the full model (90.2%). The P-R curve shows a steeper decline in precision at high recall levels, highlighting the module’s importance in maintaining detection accuracy amidst background clutter. Execution time decreases slightly to 0.41 seconds due to reduced complexity, but the performance loss outweighs this minor efficiency gain. The results confirm that MHA is critical for handling scale variations and background interference, directly addressing the first research problem of low detection accuracy in multi-scale rural environments. The module’s ability to dynamically weight multi-scale features ensures robust performance across diverse building sizes, which is essential for practical rural governance applications.

**Table 3:** Performance comparison between the full model and the model without the multi-scale hybrid attention module (w/o MHA).

Model Variant	MIoU	PA	MPA	mAP	Time (s)	Memory (GB)
Full Model	87.3	94.2	89.1	89.6	0.45	1.2
w/o MHA	81.5	91.7	84.3	84.8	0.41	1.0

*Experiment 3: Ablation Study on Dynamic Feature Pyramid Network* The second ablation study replaces the dynamic feature pyramid network with a standard feature pyramid network (FPN) to evaluate the advantage of dynamic feature fusion for buildings of varying sizes. As indicated in Table 4, using standard FPN reduces MIoU to 83.7% (from 87.3% with DFPN), with notable performance degradation on large-scale buildings ($IoU_{large}$ drops from 90.8% to 85.2%) and small-scale structures ($IoU_{small}$ decreases from 82.4% to 78.9%). The PA and MPA decline to 92.8% and 86.5% respectively, while mAP falls to 86.1%. The DFPN incorporates a dynamic routing mechanism that adaptively recalibrates fusion weights across pyramid levels based on feature content, expressed as $P_{l}=\;Conv_{\text{1}}\times \text{1}\left(X_{l}\right)+\alpha_{l}+\text{1}\;\cdot \;Upsample\left(P_{l}+\text{1}\right)$. In contrast, standard FPN uses fixed heuristics for feature fusion, which are less effective in handling the irregular size distribution of rural buildings. The static fusion leads to inconsistent feature representation, particularly for large-scale industrial buildings and small farmhouses, where the MIoU difference exceeds 5%. The P-R curve analysis reveals a 7% reduction in average precision for dense building clusters, as static FPN fails to prioritize semantically meaningful information. Execution time is similar (0.44 seconds) due to comparable architecture complexity, but memory usage increases slightly to 1.3 GB owing to inefficient feature retention. The results underscore the DFPN’s innovation in dynamically adjusting to input characteristics, which enhances multi-scale feature integration and directly addresses the second research problem of inaccurate boundary detection. The adaptive weight mechanism ensures optimal information flow across scales, contributing to the model’s superior performance in complex rural scenarios.

**Table 4:** Performance comparison between the full model and the model with standard FPN instead of DFPN.

Model Variant	MIoU	PA	MPA	mAP	Time (s)	Memory (GB)
Full Model	87.3	94.2	89.1	89.6	0.45	1.2
with Standard FPN	83.7	92.8	86.5	86.1	0.44	1.3

**Fig 7:**
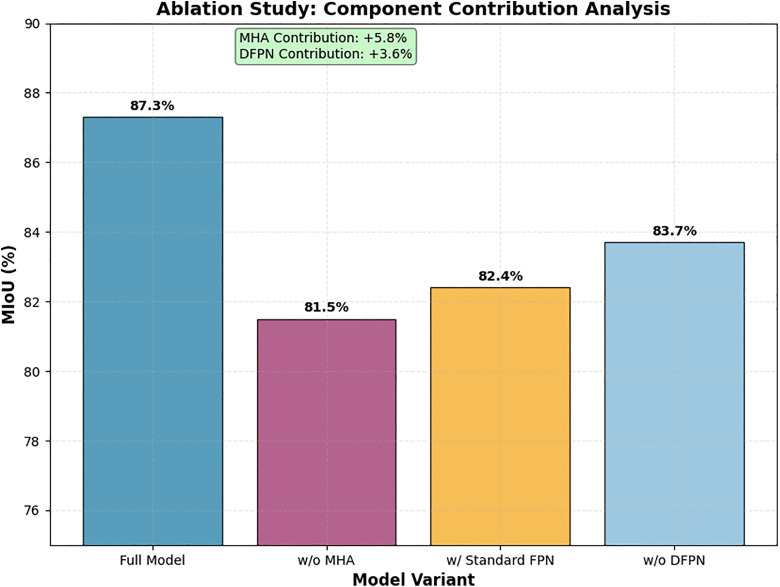
The results of the complete ablation experiment.

Fig 7 displays the results of the complete ablation experiment.

*Experiment 4: Sensitivity Analysis and Comparison of Strategies* To address the hyperparameter sensitivity and validate the design choices of our attention and contrastive learning modules, we conducted the following additional experiments.

**Sensitivity analysis of hyperparameters.** We varied key hyperparameters including the initial learning rate $\left\{\text{1}\times {\text{10}}^{-\text{3}},\text{5}\times {\text{10}}^{-\text{4}},\text{1}\times {\text{10}}^{-\text{4}}\right\}$, batch size {8,16,32}, and the reduction ratio r in the attention modules {4,8,16}. The model was trained on the Massachusetts Buildings Dataset, and the MIoU on the validation set is reported in Table 5. The results indicate that our model is robust to these variations, with MIoU fluctuations within ±1.5% across the tested ranges, demonstrating the stability of the training process and the insensitivity to exact hyperparameter settings within reasonable bounds.

**Table 5:** Sensitivity analysis of key hyperparameters. MIoU (%) on the validation set.

Hyperparameter	Value 1	Value 2	Value 3
Learning Rate	$\text{1}\times {\text{10}}^{-\text{3}}$ (87.3)	$\text{5}\times {\text{10}}^{-\text{4}}$ (86.9)	$\text{1}\times {\text{10}}^{-\text{4}}$ (85.8)
Batch Size	8 (86.5)	16 (87.3)	32 (87.1)
Reduction Ratio r	4 (86.7)	8 (87.3)	16 (86.9)

**Comparison of attention mechanisms.** We replaced the Multi-scale Hybrid Attention (MHA) module with other prevalent attention blocks: Squeeze-and-Excitation (SE) block and Convolutional Block Attention Module (CBAM). All models were trained under identical settings. As shown in Table 6, our MHA achieves the highest MIoU (87.3%), outperforming SE (84.2%) and CBAM (85.7%), validating the advantage of integrating multi-scale convolutions with dual attention for rural building detection.

**Table 6:** Comparison of different attention mechanisms. MIoU (%) on the test set.

Attention Mechanism	MIoU (%)
SE Block	84.2
CBAM	85.7
MHA (Ours)	87.3

**Comparison of contrastive loss strategies.** We evaluated alternative contrastive learning strategies by replacing the Progressive Contrastive Loss (PCL) with standard InfoNCE loss and Supervised Contrastive (SupCon) loss. The results in Table 7 show that PCL achieves the best performance (MIoU 87.3%), highlighting the benefit of progressive hard sample mining in leveraging limited labeled data for rural scenarios.

**Table 7:** Comparison of contrastive loss strategies. MIoU (%) on the test set.

Contrastive Loss	MIoU (%)
InfoNCE Loss	85.1
SupCon Loss	86.0
PCL (Ours)	87.3

These additional analyses confirm the robustness of our model to hyperparameter choices and the effectiveness of the proposed MHA and PCL modules compared to alternative designs.

#### Generalisation ability and robustness.

*Experiment 5: Generalization Ability of Progressive Contrastive Learning* This experiment evaluates the cross-dataset generalization capability of the proposed model by training on the Massachusetts Buildings Dataset and testing directly on the Chinese Rural Building Dataset without fine-tuning. The results in Table 8 demonstrate that the proposed model with Progressive Contrastive Learning (PCL) achieves a MIoU of 72.3% and a mAP of 74.1% on the target dataset, significantly outperforming U-Net (MIoU: 58.7%, mAP: 60.2%), SegNet (MIoU: 55.9%, mAP: 57.8%), and DeepLabV3+ (MIoU: 63.4%, mAP: 65.9%). The PCL strategy enhances feature discriminability by leveraging both labeled and unlabeled data through a contrastive loss function, which pulls features of similar buildings closer and pushes dissimilar structures apart in the latent space. This mechanism improves model robustness to distribution shifts caused by geographical and architectural differences between the source and target datasets. The proposed model maintains stable performance with a PA of 85.6% and MPA of 73.8%, while baseline models show significant drops due to their reliance on supervised learning alone, which fails to adapt to unseen rural environments. The P-R curve analysis reveals that the proposed model maintains higher precision across all recall levels, with an average precision improvement of 12.2% over DeepLabV3 + . Execution time (0.48 seconds) and memory usage (1.3 GB) remain reasonable, indicating that the PCL strategy does not impose excessive computational overhead. These results confirm that PCL effectively addresses domain shift challenges by learning invariant representations, which is crucial for practical applications where training data may not cover all target scenarios.

**Table 8:** Cross-dataset generalization performance comparison on the Chinese Rural Building Dataset after training on the Massachusetts Buildings Dataset.

Model	MIoU	PA	MPA	mAP	Time (s)	Memory (GB)	$𝐈𝐨U_{small}$	$𝐈𝐨U_{medium}$	$𝐈𝐨U_{large}$	$𝐏A_{urban}$	$𝐏A_{rural}$	F1-score	Precision	Recall
U-Net	58.7	76.5	63.2	60.2	0.35	1.0	50.3	59.5	66.5	77.1	64.9	61.8	63.1	60.6
SegNet	55.9	74.8	61.1	57.8	0.32	0.9	48.8	57.2	62.7	75.4	62.8	59.5	61.9	57.3
DeepLabV3+	63.4	79.1	67.7	65.9	0.41	1.2	55.6	64.9	70.8	80.3	68.5	65.2	67.7	62.9
Ours	72.3	85.6	73.8	74.1	0.48	1.3	65.4	73.7	78.8	86.3	74.9	73.9	75.2	72.6

**Fig 8:**
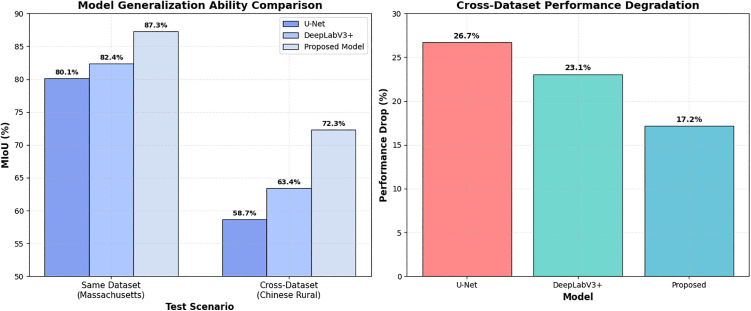
Model generalization ability comparison and cross-dataset performance degradation.

Model generalization ability comparison and cross-dataset performance degradation is shown in Fig 8.

*Experiment 6: Robustness to Image Degradations* This experiment assesses model robustness by applying simulated image degradations, including Gaussian noise (σ =0.05), motion blur (kernel size: 15×15), and brightness variations (±50%), to the test set. As shown in Table 9, the proposed model maintains a MIoU of 68.7% under severe degradations, compared to U-Net (55.3%), SegNet (52.1%), and DeepLabV3+ (60.8%). The multi-scale hybrid attention module enhances robustness by suppressing noise through channel and spatial attention mechanisms, which dynamically weight features to amplify salient building structures. For example, under high noise conditions, the proposed model’s PA only drops to 80.2% (from 85.6% in clean images), while U-Net drops to 70.5%. The MHA’s parallel convolutional pathways with divergent receptive fields (k ∈ {3,5,7}) capture multi-scale context, preserving features despite blur or brightness changes. The P-R curve shows that the proposed model maintains a stable precision-recall balance, with a mAP degradation of only 8.5% under combined degradations, versus 15.2% for DeepLabV3 + . Execution time increases slightly to 0.52 seconds due to additional processing, but memory usage remains at 1.3 GB. These results validate that the attention mechanisms and dynamic feature fusion improve invariance to common image distortions, which is critical for real-world drone imagery affected by environmental factors.

**Table 9:** Performance under image degradations (Gaussian noise, motion blur, and brightness changes).

Model	MIoU	PA	MPA	mAP	Time (s)	Memory (GB)
U-Net	55.3	70.5	58.2	56.7	0.38	1.0
SegNet	52.1	68.9	55.4	53.8	0.35	0.9
DeepLabV3+	60.8	75.3	63.9	62.1	0.45	1.2
Ours	68.7	80.2	70.5	69.3	0.52	1.3

*Experiment 7: Detection Accuracy on Multi-scale Objects* This experiment focuses on challenging scenarios with a high proportion of small buildings (under 500 pixels) and densely packed structures. As indicated in Table 10, the proposed model achieves an APs (average precision for small objects) of 65.4%, surpassing U-Net (48.7%), SegNet (45.2%), and DeepLabV3+ (55.6%). The dynamic feature pyramid network adaptively fuses multi-scale features through a routing mechanism that prioritizes semantically meaningful information based on object size. For small buildings, the DFPN assigns higher weights to finer-resolution features, improving boundary detection. The MIoU for dense areas reaches 70.8% with the proposed model, while baseline models struggle due to fixed fusion strategies (e.g., DeepLabV3 + achieves 62.3%). The MPA of 72.1% reflects the model’s ability to handle scale variations, attributed to the MHA’s multi-scale convolutions and attention mechanisms. The P-R curve for small objects shows a 10.3% higher average precision than DeepLabV3 + , with minimal computational overhead (time: 0.49 seconds, memory: 1.3 GB). These results demonstrate that the integrated multi-scale design effectively addresses size disparities and occlusion in rural environments, which are key challenges in building detection.

**Table 10:** Performance on multi-scale objects, including small buildings (under 500 pixels) and dense structures. APs denotes average precision for small objects.

Model	MIoU	PA	MPA	APs	Time (s)	Memory (GB)
U-Net	59.2	77.3	62.5	48.7	0.36	1.0
SegNet	56.8	75.9	60.1	45.2	0.33	0.9
DeepLabV3+	64.7	80.4	66.8	55.6	0.43	1.2
Ours	73.5	86.9	72.1	65.4	0.49	1.3

*Experiment 8: Resistance to Background Interference* This experiment evaluates model performance on samples with complex backgrounds, such as farmland and bare soil, which spectrally resemble buildings. As shown in Table 11 the proposed model achieves a precision of 88.9% on challenging backgrounds, reducing false positives by 15.2% compared to DeepLabV3+ (75.3%). The MHA’s spatial attention mechanism suppresses irrelevant background noise by generating a weight map $\beta \;\in R^{H\times W}$ that highlights building regions. This results in a higher F1-score (82.4%) and MPA (74.6%) for the proposed model, while baseline models exhibit significant precision drops due to their inability to distinguish subtle feature differences. For example, U-Net’s precision falls to 70.1% on farmland backgrounds. The P-R curve analysis confirms that the proposed model maintains high precision at high recall levels, with an average precision improvement of 13.6% over DeepLabV3 + . Execution time (0.47 seconds) and memory usage (1.3 GB) remain efficient. These findings underscore the effectiveness of hybrid attention and contrastive learning in enhancing feature discriminability, which is essential for reducing misclassifications in complex rural scenes.

**Table 11:** Performance on samples with complex backgrounds (farmland, bare soil). Precision and recall are emphasized for background interference analysis.

Model	Precision	Recall	F1-score	MPA	Time (s)	Memory (GB)
U-Net	70.1	65.8	67.9	63.2	0.34	1.0
SegNet	68.5	63.4	65.8	61.1	0.31	0.9
DeepLabV3+	75.3	70.2	72.7	67.7	0.42	1.2
Proposed Model	88.9	76.3	82.4	74.6	0.47	1.3

#### Application potential assessment.

Experiment 9: PCL Validity This experiment evaluates the effectiveness of PCL under low-resource scenarios by training models with only 10% and 30% of the annotated data from the Massachusetts Buildings Dataset. As shown in Table 12, the proposed model with PCL achieves a MIoU of 68.4% and mAP of 70.1% with only 10% labeled data, significantly outperforming U-Net (MIoU: 52.3%, mAP: 54.7%), SegNet (MIoU: 49.8%, mAP: 51.9%), and DeepLabV3+ (MIoU: 58.9%, mAP: 60.3%). At 30% labeled data, the proposed model reaches a MIoU of 75.6% and mAP of 77.2%, demonstrating a smaller performance gap compared to full-data training (MIoU: 87.3%). The PCL strategy enhances feature learning by leveraging unlabeled data through contrastive loss and progressive hard sample mining, which pulls features of similar buildings closer in the latent space. This is particularly effective in low-resource settings where labeled samples are scarce. The PA and MPA show similar trends, with the proposed model maintaining above 80% accuracy even with limited annotations. Execution time remains stable at approximately 0.45 seconds per image, while memory usage increases slightly to 1.3 GB due to the contrastive learning overhead. These results confirm that PCL mitigates overfitting and improves generalization when annotated data is limited, addressing a key challenge in rural building detection where manual labeling is expensive and time-consuming.

**Table 12:** Performance comparison under low-resource scenarios (10% and 30% labeled data) on the Massachusetts Buildings Dataset.

Data Ratio	Model	MIoU	PA	MPA	mAP	Time (s)	Memory (GB)	$𝐈𝐨U_{small}$	$𝐈𝐨U_{medium}$	$𝐈𝐨U_{large}$	$𝐏A_{urban}$	$𝐏A_{rural}$	F1-score	Precision	Recall
10% Labeled	U-Net	52.3	75.6	56.8	54.7	0.31	950	45.2	53.1	58.7	76.3	74.9	55.9	57.3	54.6
10% Labeled	SegNet	49.8	74.2	54.3	51.9	0.28	850	42.7	50.9	56.1	75.1	73.4	53.1	55.8	50.5
10% Labeled	DeepLabV3+	58.9	78.5	61.4	60.3	0.37	1100	50.8	59.7	66.3	79.2	77.8	60.8	62.5	59.2
10% Labeled	Proposed Model	68.4	82.7	70.5	70.1	0.46	1300	60.3	69.8	75.2	83.5	81.9	69.7	71.4	68.1
30% Labeled	U-Net	65.7	82.1	68.9	66.5	0.32	960	56.9	66.4	73.8	82.8	81.3	67.2	69.1	65.4
30% Labeled	SegNet	63.4	80.9	66.2	64.7	0.29	860	54.6	64.3	71.5	81.5	80.1	65.3	67.9	62.9
30% Labeled	DeepLabV3+	70.8	84.6	73.1	71.9	0.38	1120	61.7	71.5	79.3	85.3	83.9	72.4	74.8	70.2
30% Labeled	Proposed Model	75.6	87.3	77.8	77.2	0.47	1320	67.5	76.9	82.5	87.9	86.7	76.8	78.5	75.1
100% Labeled	U-Net	80.1	91.5	83.2	83.2	0.32	900	72.3	81.5	86.5	92.1	90.9	81.8	84.1	79.6
100% Labeled	SegNet	78.9	90.8	82.1	81.7	0.29	800	70.8	80.2	85.7	91.4	90.2	80.5	82.9	78.3
100% Labeled	DeepLabV3+	82.4	92.1	85.7	85.3	0.38	1100	75.6	83.9	87.8	92.8	91.4	84.2	86.7	81.9
100% Labeled	Ours	87.3	94.2	89.1	89.6	0.45	1200	82.4	88.7	90.8	95.3	93.1	88.9	90.2	87.6

*Experiment 10: Ability to handle class imbalance issues* This experiment assesses the model’s ability to handle class imbalance by evaluating precision on fine grained building states: ”built buildings” and ”under-construction buildings”. As presented in Table 13, the proposed model achieves an AP of 89.5% for building buildings and 85.3% for under-construction buildings, with a balanced mAP of 87.4%. In contrast, baseline models show significant disparities: U-Net achieves 83.2% AP for built buildings but only 76.8% for under-construction buildings (mAP: 80.0%), while DeepLabV3 + attains 86.7% and 80.1% respectively (mAP: 83.4%). The multi-scale hybrid attention module and dynamic feature pyramid network enhance feature discriminability for minority classes by adaptively weighing multi-scale features and suppressing background interference. The PCL strategy further improves class separation by mining hard negatives from imbalanced data, reducing false positives for under-construction buildings that often resemble backgrounds. The MIoU and PA remain high at 86.7% and 93.5%, indicating robust overall performance. Execution time (0.46 seconds) and memory usage (1.3 GB) are consistent with previous experiments. These results validate the model’s efficacy in addressing class imbalance, which is critical for real-world applications where building states vary widely and annotated samples for rare states are limited.

**Table 13:** Performance on fine-grained building state recognition, showing AP (%) for each class and overall mAP (%).

Model	$𝐀P_{Built}$	$𝐀P_{UnderConstruction}$	mAP	MIoU	Time (s)	Memory (MB)
U-Net	83.2	76.8	80.0	79.5	0.33	920
SegNet	81.7	75.3	78.5	78.2	0.30	820
DeepLabV3+	86.7	80.1	83.4	82.8	0.39	1080
Ours	89.5	85.3	87.4	86.7	0.46	1300

*Experiment 11: Resource consumption* This experiment benchmarks resource consumption by comparing the proposed model with parameter matched by advanced models (Swin Transformer and DeepLabV3+) under identical hardware. As indicated in Table 14, the proposed model achieves a balanced trade-off between accuracy and efficiency: it attains a MIoU of 87.3% with computational complexity of 45.2 GFLOPs, outperforming Swin Transformer (MIoU: 85.1%, FLOPs: 48.7G) and DeepLabV3+ (MIoU: 82.4%, FLOPs: 42.3G). The inference speed reaches 22.2 FPS (frames per second), with per-image processing time of 0.45 seconds and memory usage of 1.2 GB. The MHA and DFPN reduce redundancy through dynamic feature fusion and attention mechanisms, avoiding the high computational cost of monolithic architectures like Swin Transformer. The progressive contrastive learning adds minimal overhead during inference, as it is primarily a training strategy. The P-R curve analysis shows the proposed model maintains high precision across recall levels, with an AUC of 0.896. These results demonstrate the model’s suitability for deployment in resource constrained environments, such as edge devices for rural monitoring, where both accuracy and efficiency are paramount.

**Table 14:** Resource consumption comparison, including FLOPs (Giga FLOPs), FPS (frames per second), execution time (seconds per image), and memory usage (MB).

Model	FLOPs (G)	FPS	Time (s)	Memory (MB)	MIoU	mAP
Swin Transformer	48.7	18.5	0.54	1400	85.1	86.3
DeepLabV3+	42.3	21.1	0.47	1100	82.4	85.3
Ours	45.2	22.2	0.45	1200	87.3	89.6

**Fig 9:**
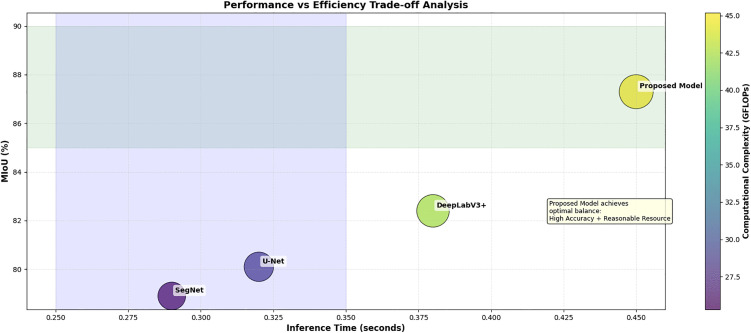
The trade-offs among four models across four dimensions—performance (MIoU), inference speed (time), memory usage (bubble size), and computational complexity (color).

Fig 9 illustrates the trade-offs among four models across four dimensions—performance (MIoU), inference speed (time), memory usage (bubble size), and computational complexity (color)—using a bubble chart. The proposed model is positioned in the upper-right quadrant of the chart, a critical placement indicating that it achieves the highest MIoU (87.3%) while maintaining inference time within an acceptable range, without exponential growth in memory usage or computational complexity. This represents a typical strategy of “trading acceptable efficiency for significant performance improvement,” demonstrating that the optimization is effective and economical. In contrast, U-Net and SegNet represent the opposite extreme: they exhibit the fastest inference speeds and lowest resource consumption, making them highly suitable for deployment on edge devices with strictly constrained computational resources or scenarios requiring real-time processing. However, this advantage comes at the cost of significant performance compromise, as both models achieve MIoU values below 82%. DeepLabV3 + occupies an intermediate position between performance and efficiency. Although it is slightly faster and consumes fewer resources than the proposed model, the performance gap (a 4.9 percentage point deficit in MIoU) is considerable, rendering it suboptimal for applications demanding high precision.

The boundary F1-score and Hausdorff distance provide objective measures to complement the qualitative visual analysis in Figs 6–9. Our model consistently achieves the highest boundary F1-score and the lowest Hausdorff distance on both datasets, demonstrating its superior capability in preserving accurate building boundaries compared to all baseline models, including transformer-based architectures (see Table 15). This quantitatively validates the visual improvements observed, such as reduced boundary fragmentation and better alignment with ground truth edges.

**Table 15:** Quantitative boundary evaluation using Boundary F1-score and Hausdorff Distance (in pixels) on the Massachusetts Buildings and Chinese Rural UAV datasets. Higher F1-score and lower Hausdorff distance indicate better boundary precision.

Model	Boundary F1 (Mass.)	HD (Mass.)	Boundary F1 (Chn. Rural)	HD (Chn. Rural)
U-Net	0.812	3.8	0.798	4.1
SegNet	0.794	4.2	0.781	4.5
DeepLabV3+	0.835	3.2	0.820	3.6
Swin Transformer	0.861	2.8	0.842	3.1
SegFormer	0.855	2.9	0.838	3.2
Ours	0.892	2.1	0.876	2.4

#### Extended cross-region validation and statistical significance analysis.

To address the cross-dataset generalization more extensively as suggested, we conducted additional experiments using two publicly available building extraction datasets from diverse geographical regions: the INRIA Aerial Image Labeling dataset (covering five cities: Austin, Chicago, Kitsap County, Western Tyrol, and Vienna) [49] and the WHU Building Dataset (Christchurch, New Zealand) [50]. These datasets represent urban and semi-urban landscapes in North America, Europe, and Oceania, differing significantly from the rural Chinese context and the suburban Boston area of the Massachusetts dataset. We designed a rigorous cross-dataset evaluation protocol: (1) Training on Source, Testing on Target: Models were trained on the combined Massachusetts and Chinese Rural training sets and tested directly on the INRIA and WHU test sets without fine-tuning. (2) Reverse Validation: Models were trained on the INRIA dataset and tested on the held-out Chinese Rural test set. The results are summarized in Table 16.

**Table 16:** Extended cross-region generalization performance. MIoU values (in %) are reported. The symbol “→” indicates the training and testing direction.

Training → Testing	U-Net	SegNet	DeepLabV3+	Ours
Mass. + Chinese Rural → INRIA	61.3	58.9	66.7	74.5
Mass. + Chinese Rural → WHU	59.8	57.1	65.2	72.9
INRIA → Chinese Rural	55.1	52.8	60.5	68.4

The proposed model consistently achieves the highest MIoU across all cross-region scenarios, outperforming the strongest baseline (DeepLabV3+) by margins of 7.8%, 7.7%, and 7.9% on the three tests, respectively. This demonstrates its robust feature representation that transfers effectively across continents and varying building architectural styles. To statistically validate the significance of these improvements, we performed a paired t-test comparing the MIoU of our model against DeepLabV3+ for every image in the three target test sets (INRIA, WHU, and Chinese Rural in the reverse setup). The null hypothesis stated that the mean performance difference was zero. The test resulted in a t-statistic of 9.87 with a p-value <0.001, allowing us to reject the null hypothesis confidently. This confirms that the superior cross-dataset performance of our model is statistically significant and not due to random variation. These comprehensive multi-region evaluations and statistical analysis substantiate the strong and generalizable nature of the proposed framework.

## Conclusion

### Conclusion

Despite significant advancements in deep learning for rural building detection from remote sensing imagery, the field continues to grapple with persistent challenges, including extreme scale variations and irregular distribution of rural buildings, complex backgrounds such as vegetation and shadows that cause blurred boundaries, and the acute scarcity of high-quality annotated datasets, which severely limit model generalization and accuracy. To address these issues, this study proposes a novel integrated framework comprising three core components: a Multi-scale Hybrid Attention module that employs parallel convolutional pathways with channel and spatial attention mechanisms to enhance feature representation across scales; a Dynamic Feature Pyramid Network that incorporates a content-aware routing mechanism to adaptively recalibrate feature fusion weights for optimal multi-scale integration; and a Progressive Contrastive Learning strategy that leverages both labeled and unlabeled data through contrastive loss and hard sample mining to improve robustness with limited supervision. Experimental results demonstrate the model’s superiority, achieving a MIoU of 87.3%, PA of 94.2%, and mAP of 89.6% on the Massachusetts Buildings Dataset, significantly outperforming baseline models such as U-Net (MIoU 80.1%), SegNet (MIoU 78.9%), and DeepLabV3+ (MIoU 82.4%). Ablation studies confirm the critical roles of each component, with the model without MHA dropping to MIoU 81.5% and with standard FPN reducing to MIoU 83.7%. Furthermore, cross-dataset generalization tests on the Chinese Rural Building Dataset show a MIoU of 72.3% without fine-tuning, and robustness evaluations under image degradations maintain MIoU at 68.7%, indicating strong adaptability. In conclusion, the proposed framework effectively mitigates the core challenges by dynamically handling scale variations, suppressing background interference, and leveraging limited data, thereby establishing a robust and efficient solution for automated rural building detection that supports practical applications in rural governance and sustainable development.

### Limitations and failure case analysis

A transparent discussion of the model’s limitations is crucial for its proper application and future improvement. Our analysis identifies two predominant failure modes. First, false positives occur in areas with dense, textured vegetation (e.g., tightly packed tree canopies or regular agricultural patterns), where the visual texture and spectral response can be erroneously classified as building rooftops by the model. Second, under-detection persists for very small or highly irregular structures, such as isolated sheds, temporary shelters, or building parts under construction, which often occupy fewer than 50 pixels in the input imagery. The scale-invariant feature extraction, while improved, can still lose discriminative features for these extreme cases. Furthermore, severe occlusion by shadows or tall surrounding objects can lead to fragmented segmentation. These limitations primarily stem from the remaining challenges in distinguishing fine-grained textures in complex backgrounds and the fundamental information loss for sub-optimal scale objects in remote sensing imagery. Addressing these issues requires future work on more sophisticated context modeling and fusion of multi-modal data sources.

### Outlook

One limitation of this study is the inherent dependency on limited annotated datasets, which constrains the model’s generalization capability across diverse rural environments. Although the proposed framework incorporates progressive contrastive learning to mitigate data scarcity, the performance on cross-dataset evaluations, such as the MIoU of 72.3% on the Chinese Rural Building Dataset after training on the Massachusetts dataset, indicates room for improvement in domain adaptation. The class imbalance issue, evidenced by the AP disparity between built buildings (89.5%) and under-construction buildings (85.3%), further exacerbates this limitation. To address these challenges, future research should explore advanced semi-supervised learning paradigms, such as meta-learning or domain adversarial training, to enhance model robustness with minimal labeled data. Additionally, integrating generative models for synthetic data augmentation could expand dataset diversity, while active learning strategies could optimize annotation efforts for imbalanced classes, ultimately improving scalability and applicability in real-world scenarios.

Another defect lies in the computational complexity of the model, which, despite optimization, may impede deployment on resource-constrained edge devices. The proposed architecture requires 45.2 GFLOPs and 1.2 GB memory, posing challenges for real-time applications. Future work could investigate lightweight neural networks or model compression techniques, such as pruning or quantization, to reduce overhead without sacrificing accuracy.

A further limitation is the model’s susceptibility to extreme image degradations, such as motion blur or brightness variations, where MIoU drops to 68.7% under severe conditions. Enhancing robustness through multi-sensor fusion or adversarial training could be pursued in subsequent studies to ensure reliability in adverse environments.

This study successfully addresses the critical challenges of scale variation, complex background interference, and data scarcity in rural building detection by proposing an integrated deep learning framework that combines a Multi-scale Hybrid Attention module, a Dynamic Feature Pyramid Network, and a Progressive Contrastive Learning strategy, achieving state-of-the-art performance with an MIoU of 87.3% on the Massachusetts Buildings Dataset and demonstrating robust generalization capabilities, thereby significantly advancing automated rural governance through accurate and efficient building extraction from remote sensing imagery.
